# A Warm Welcome to the Diagnostic and Statistical Manual of Mental Disorders, Fifth Edition?

**Published:** 2014

**Authors:** Emran Mohammad Razzaghi

**Affiliations:** 1 Associate Professor, Department of Psychiatry, School of Medicine, Tehran University of Medical Sciences, Tehran, Iran.

## Abstract

The present article discusses the reaction of psychiatric professionals and specifically the academia in Iran to the publication of the Diagnostic and Statistical Manual of Mental Disorders, Fifth Edition, (DSM-5). It is argued that the reaction to changes and the approach of the new revision of DSM has been neither technical, nor emotional. This argument has further been examined for the previous revisions of the DSM and the conclusion is that the Iranian psychiatry has become alienated from the need and necessity of classification of mental disorders from a local and national point of view. The reason for this alienation has further been discussed and has been focused on the lack of contact between the psychiatric catchment areas and the academia.

**Declaration of interest:** None.

“The new version of DSM was released in May 2013 following a long process of planning and research stages that date back to 1999 (1). The open challenge however, has not prevented the academia and professionals from expression of their concerns and criticism regarding the final output of the marathon. The bold opposition of Dr. Thomas Insel, Director of the US National Institute on Mental Health (NIMH) and the response from Dr. David Kupfer, the Chair of APA’s DSM-5 Task Force has made it clear that reactions to an overhaul to the “Bible” will not be conservative (2,3).

In Iran’s professional milieu, however, I have not seen any opposition statement or response, so far. Although, with a total number of around 1500 psychiatrists - 2 per 100,000 population - and 9000 psychologists - PhD, MSc, and BSc holders, altogether - the silence sounds intriguing! I was trained as a psychiatrist under DSM-III, and have witnessed the introduction of the III-R, IV, and IV-TR versions prior to this recent move. While there has been a similar “blunted affect” to the changes among the Iranian mental health professionals, still I recall that in the 1980s it was not uncommon to hear judgments about the evolution of DSM from Iranian key figures such as the late Dr. Haritun Davidian and Dr. Gholamreza Bahrami, but this happened to fade out in the following years. The prominence of the event for us, however, is the absolute dominance of DSM over the ICD in Iranian psychiatry. In other words, it would not be an exaggeration to say that the Iranian psychiatry’s reaction to the distortion of its Bible has been “neutral”. In this article I would like to analyze and discuss the situation from a constructive view.

In recent years there has been a substantial rise in the number of publications in the domain of psychiatry in Iran. Thus, a paucity of publications or tendency to question and measure events and phenomena may be out of consideration. The inconsistency, however, might be a sign of basic and methodological flaws in the process of science production in Iran. In other words, most publications are limited to plain observations, rather than searching for answers to shortcomings and unsolved conditions, not mentioning the lack of introduction of hypotheses and the testing of their validity. In search for the precipitating and perpetuating factors to this condition, I would preferably differentiate the existing situation from the ideal scenario that is under examination in this article, by the analogy of superficial versus deep and profound. Keeping in mind my observation that a few decades back there was deep questioning of the classification of mental disorders, it is by no means straightforward to think of a professional and scientific decline in recent years despite the doubling of the number of psychiatrists in the same period. I would rather try to examine it from the angle of comprehensiveness of Iranian psychiatrists in the recent past, and would identify it as the declining element here. Again, would this diminishing comprehensiveness be a result of a downward slope in psychiatric residency training? I would rather disagree. Not counting the individual departments of psychiatry training initiatives and the hard competition between universities that have resulted in local reforms in training programs, the curriculum of psychiatric residency training was revised and extended at national level by the Ministry of Health in late 2000s and a multitude of training strategies have been added to previous training approaches in the time-period under discussion. Therefore, the current more competitive academic and training performance may rule out a direct dwindling of residency training standards as the primary reason for the “bluntness”.

For us as Iranian professionals the term “consumerism in science” is a familiar notion. Perhaps our understanding of this very term has been the motive for the current surge in research and publications. However, my argument here is that our endeavor has not led to a level of self-esteem that could lead to genuine productivity in different scientific fields nor has it led to accumulation of scientific capital to a level necessary to result in deeper science formation. The Iranian model of science consumerism, therefore, may be defined as an intermediary stage in between pure duplication and consumption of scientific production of other schools, and actual productivity with a particular native orientation. We do produce some kind of knowledge that in its best sense lacks immediate applicability to our current needs and challenges. In search to identify causes of such aberration I would focus on a specific point. We lack an appropriate mechanism to translate our questions into research. Replying to any possible critic that my point, so far, adds not much more than what many scholars have said repeatedly, by deepening my analysis I would add however, that we lack the mechanism to see the question per se.

In return to the DSM issue and our bluntness, my argument would thus be that we have not faced questions in terms of classification of mental disorders, and therefore we are neutral to others’ positions. The question, here, is that while we are practicing psychiatry on a daily basis and the number of psychiatrists has mounted during the past two decades why are we not facing questions? My answer to this important question is not complicated. We do practice psychiatry, but not on a systematized basis; being responsive to the mental health not only at individual level but also at community level. The current psychiatric practice in Iran is on a passive basis, and only responsive to demands and not to needs. As we do not follow specific and predefined mental health standards and indicators, there are no well-defined infrastructures responsible for them. For example, almost all school mental health programs are just a reflection of a mental health professional eagerness, and not a sign of demand from the school authorities. Similarly, aftercare remains an ideal luxury in our mental health service. As a psychiatrist practicing and training psychiatry in a most prestigious academic center in Iran, I have not been taken responsible for any minimum mental health standards of a “catchment” community. Nor am I involved in pre-hospital and aftercare services my patients might be in need of. I would argue then, that for this reason, I am not obliged to shift from a consumerist position to a productive one, and that is the actual reason that my position towards the DSM changes remains neutral. Classification of diseases in its broader sense, and the DSM in specific, is a tool for responding to needs at community level. Fine-tuning of definitions and criteria would affect social standards, mental health standards, and most importantly it would make adjustments to the financial capital invested in mental health. Not to mention the primitive approach to mental health insurance industry in Iran; as long as the major part of medical payments is “out of pocket” and, therefore, not “managed” in any sense, we would remain indifferent to DSM changes. No matter that the largest fraction of the health system in Iran belongs to the private sector, we need to somehow “manage” the mental health of the community. My solution in this regard is strict adherence to “managed care” principles with the departments of psychiatry in the more important universities taking a prominent role in this management by taking responsibility of the already defined - but not well respected and practiced - catchment area of their university and acting as a protective, supportive, and advisory umbrella for all the private and public sector services in fulfilling mental health standards. 

My earlier point in referring to the expression of “attitude” towards earlier versions of DSM by late figures in Iran’s psychiatry brings me to this interpretation that those figures practiced psychiatry in an era of very limited number of psychiatrists. Therefore, while even in circumstances not able to adhere to high standards, they had to remain responsible to minimal demands of the community, just because they were few and were under the pressure of their rarity and fame. To them, therefore, changes in DSM or any other classification system would mean higher or lower responsibilities in terms of classifying human behaviors and conditions as disorder, aberrant, or normal. Today, however, that the number of mental health professionals is more than can be identified and taken responsible by the community, this would be the responsibility of medical universities in general and the departments of psychiatry in specific. Having not met this responsibility, our universities have thus engaged in “fun/luxurious” research, as explained earlier. I am afraid I do not see the likelihood of any promising alternative that could assure reaching the level of having positions towards the DSM, in the prospect.
